# Supplementary material for: Phytochemical Composition, Antioxidant, Anti-Helicobacter pylori, and Enzyme Inhibitory Evaluations of Cleistocalyx operculatus Flower Bud and Leaf Fractions
Source: BioTech (Basel). 2024 Oct 11;13(4):42. doi: 10.3390/biotech13040042 (PMC11503338; doi:10.3390/biotech13040042)
Supplement: Supplementary file 1 [file biotech-13-00042-s001.zip › biotech-3177622-supplementary.pdf]

Supplementary Material

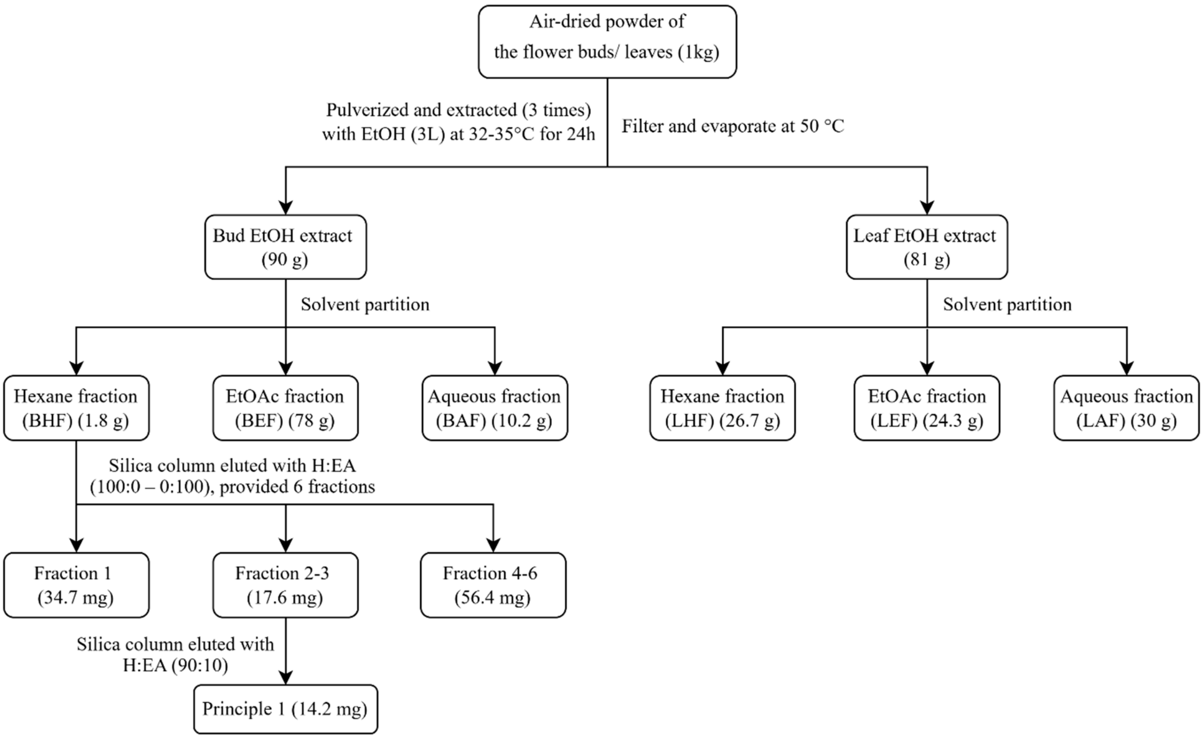

Figure S1. Scheme of flower bud and leaf solvent fractions partitioned by liquid-liquid fractionation from flower bud and leaf ethanolic extracts of *Cleistocalyx operculatus* and isolation of principle 1 derived from flower bud hexane fraction (BHF).

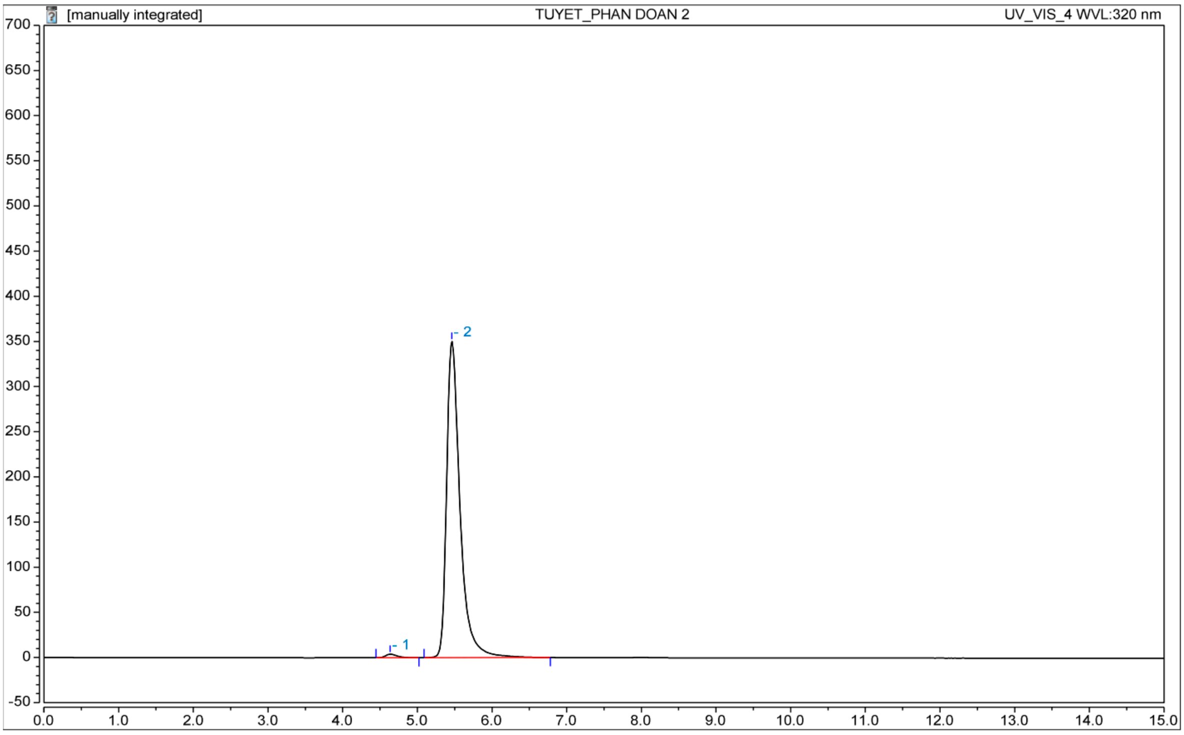

Integration Results

No.	Peak Name	Retention Time min	Area mAU*min	Height mAU	Relative Area %	Relative Height %	Amount n.a.
1		4.637	0.696	3.924	0.94	1.11	n.a.
2		5.463	73.038	350.432	99.06	98.89	n.a.
**Total:**			**73.734**	**354.356**	**100.00**	**100.00**	

Figure S2. HPLC of DMC isolated from the flower bud hexane fraction (BHF)

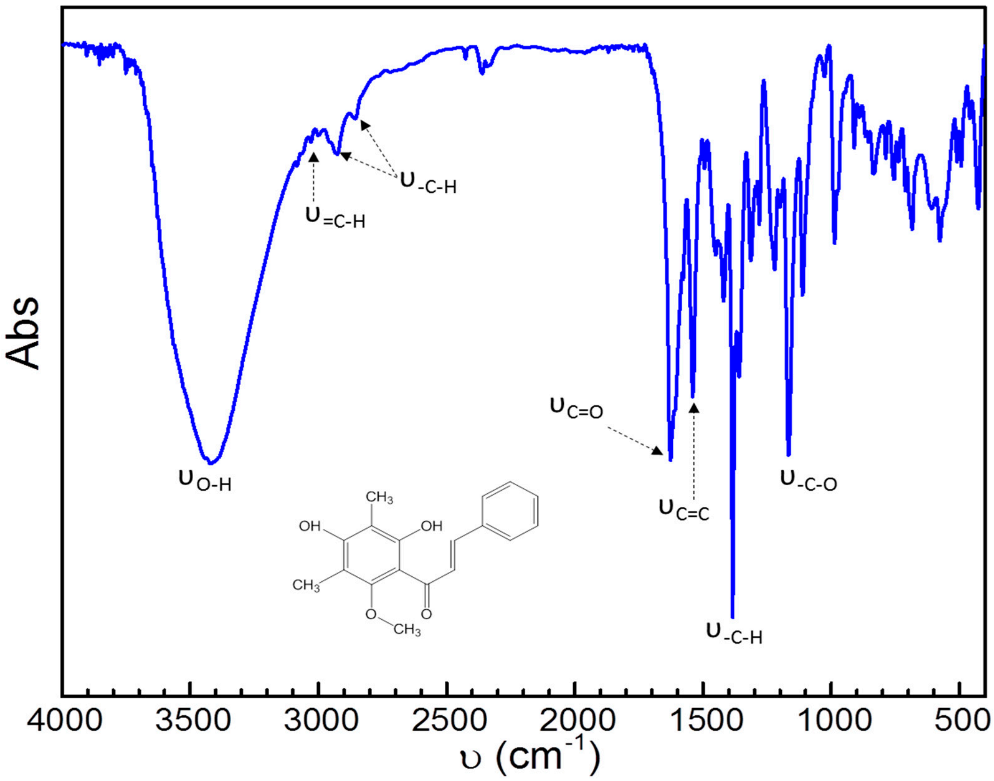

Figure S3. FT-IR data of DMC isolated from flower bud hexane fraction (BHF)

Table S1. ^{1}H NMR, ^{13}C-NMR, and HMBC data of DMC isolated from flower bud hexane fraction (BHF)

Position	^{13}C (ppm)	^{1}H (ppm, J μHz)	HMBC ^{1}H → ^{13}C
1	135.5	-	
2	129.0	7.65 (dd, 7.6, 2.0)	C-β, 3, 4, 5
3	128.7	7.41 (m)	1, 2
4	130.3	7.41 (m)	2, 3, 5, 6
5	128.7	7.41 (m)	1, 2
6	129.0	7.65 (dd, 7.6, 2.0)	C-β, 3, 4, 5
C-β	143.0	7.84 (d, 15.7)	1, 2, 6, C-α, C=O
C-α	126.9	7.99 (d, 15.7)	1, C-β, C=O
C=O	193.5	-	-
1'	109.0	-	-
2'	162.2	-	-
3'	109.2	-	-
4'	159.3	-	-
5'	106.7	-	-
6'	159.0	-	-
2'-OH	-	13.2 (s)	1', 2', 3'
3'-CH_3	8.4	2.16 (s)	2', 3', 4'
5'-CH_3	7.7	2.14 (s)	4', 5', 6'
6'-OCH_3	62.5	3.66 (s)	6'

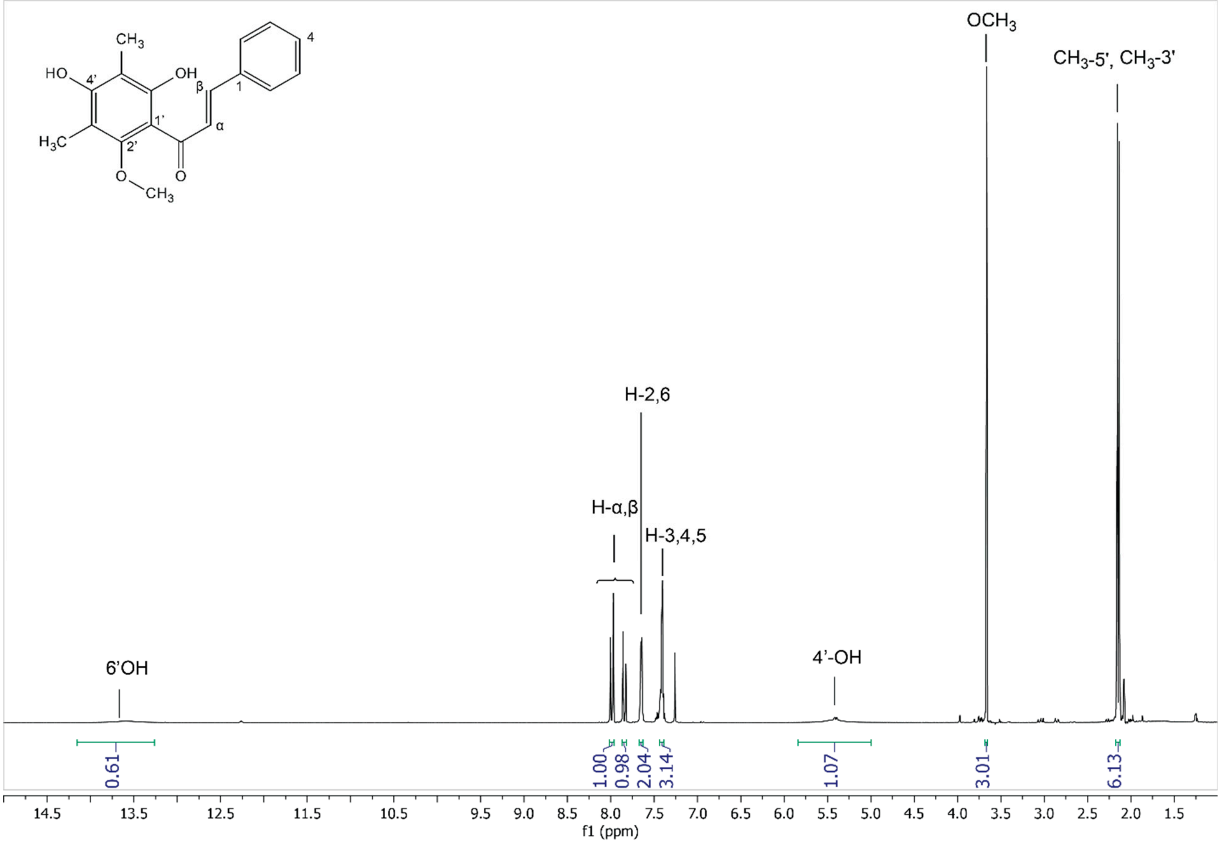

Figure S4. ^{1}H-NMR data of DMC isolated from the flower bud hexane fraction (BHF)

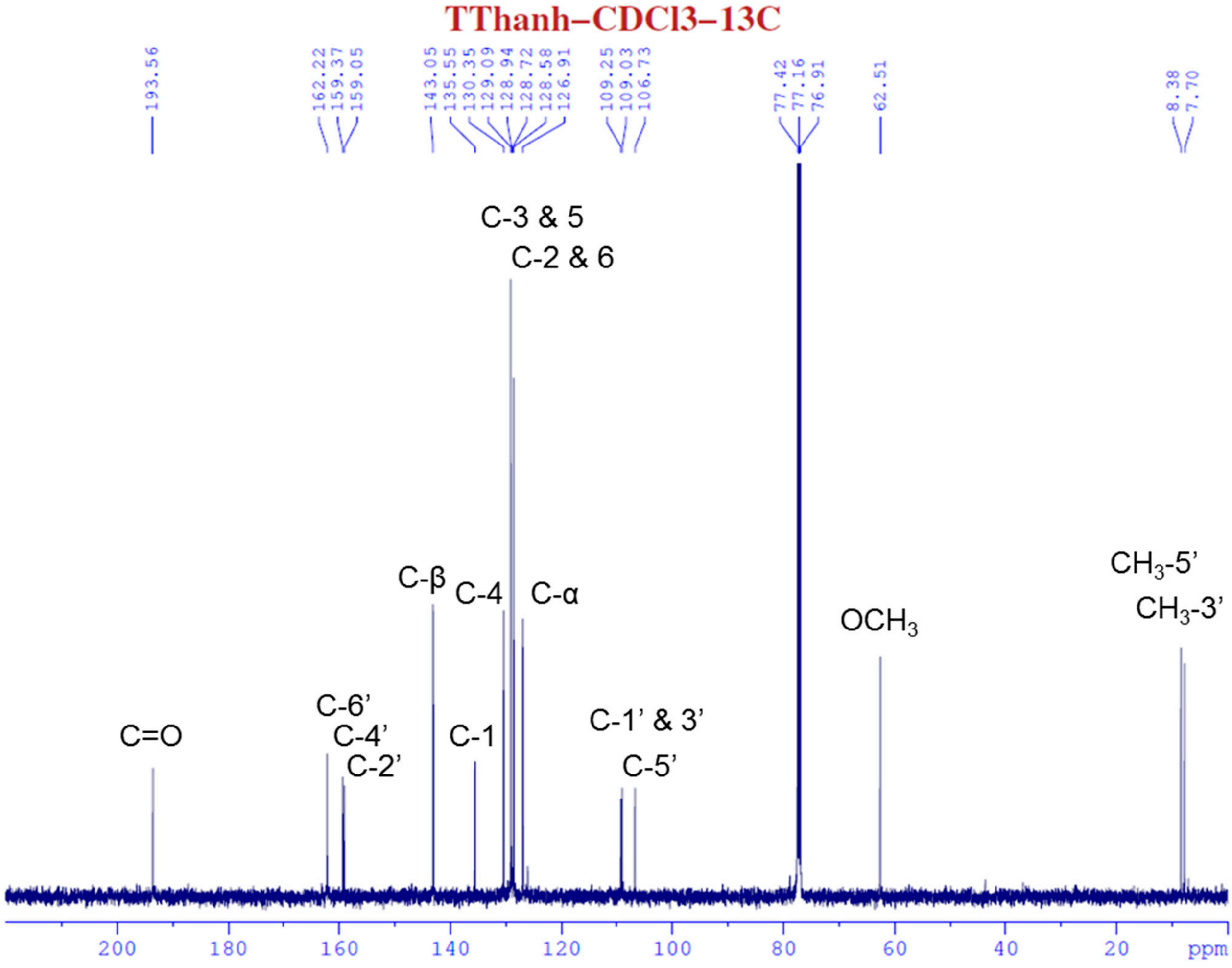

Figure S5. ^{13}C-NMR data of DMC isolated from flower bud hexane fraction (BHF)

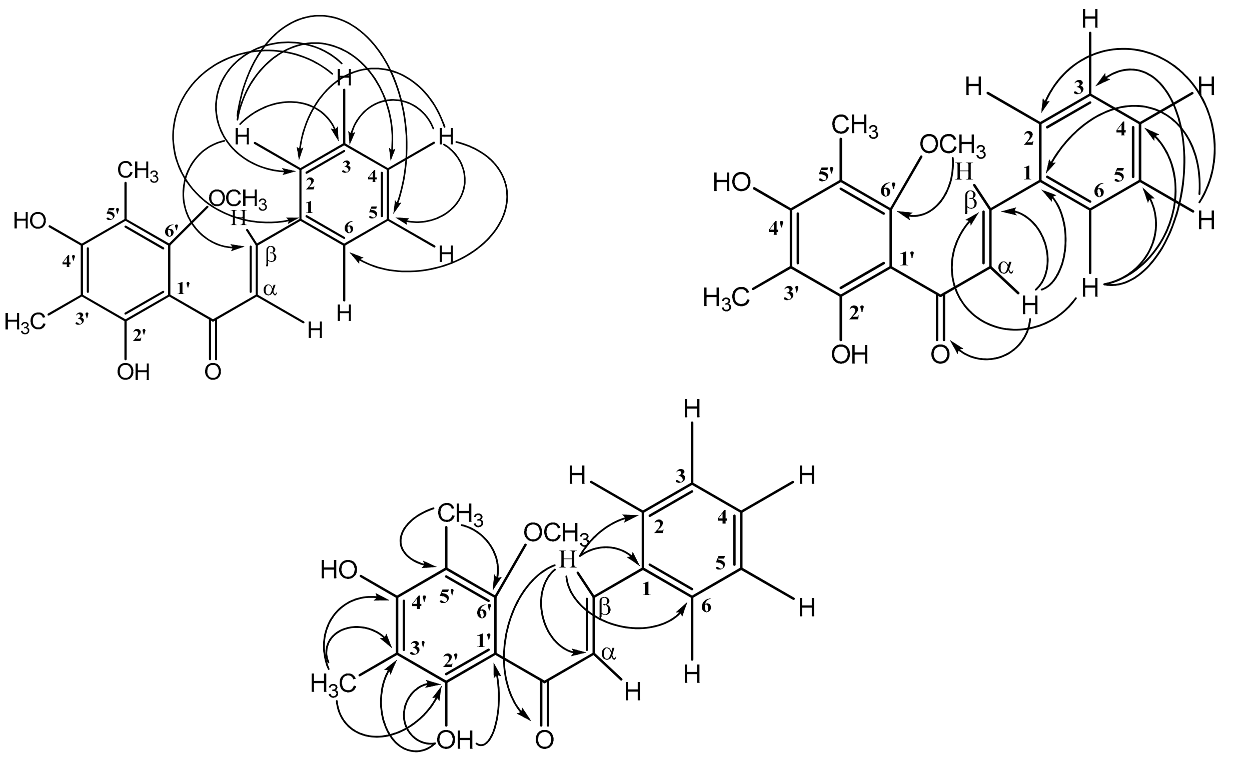

Figure S6. ^{1}H - ^{13}C HMBC-correlations of DMC isolated from flower bud hexane fraction (BHF)
